# Nanogenerators for gas sensing applications

**DOI:** 10.3389/fchem.2024.1532018

**Published:** 2025-01-10

**Authors:** Ye-Xuan Zhen, Gong Wang, Yun-Fei Li, Yu Yu

**Affiliations:** ^1^ Center for Advanced Laser Technology, Hebei University of Technology, Tianjin, China; ^2^ Innovation and Research Institute of Hebei University of Technology in Shijiazhuang, Shijiazhuang, China; ^3^ Hebei Key Laboratory of Advanced Laser Technology and Equipment, Tianjin, China

**Keywords:** piezoelectric nanogenerators, triboelectric nanogenerators, self-powered sensing system, gas sensor, gas-sensitive materials

## Abstract

Gas sensors are now widely employed in many industries due to the rapid speed of industrialization and the growth of the Internet of Things. However, the wearability and mobility of traditional gas sensors are limited by their high reliance on external power sources. Nanogenerators (NGs) can compensate for their power source limitations when paired with gas sensors by transforming the environment’s widely dispersed low-frequency energy into electrical energy, allowing for self-powered gas detection. The paper thoroughly examines the advancements made in the field of NG-based self-powered gas sensor research in recent years. A systematic description is given of the two main types of NG-based self-powered gas sensors. Lastly, the evolution of sensor use in a few typical gas sensing applications is highlighted, and the field’s future development trend is anticipated.

## 1 Introduction

A gas sensor measures the concentration and composition of a target gas. It transforms the reaction between the target gas and gas-sensitive material into a measurable electrical signal. The device’s performance is then assessed using several metrics, including sensitivity, selectivity, accuracy, recovery time, and response time (Nazemi et al., 2019). Applications of gas sensors have been widely implemented in various fields, such as agricultural production (Seesaard et al., 2022), industrial production (Yuan et al., 2022; Liu et al., 2023b), human health monitoring (Su et al., 2021), environmental monitoring (Chang et al., 2022a), and food quality assessment (Andre et al., 2022). The working principles of a variety of gas sensors, such as resistive (Majhi et al., 2021), capacitive (Wu et al., 2022), optical (Dinh et al., 2016), *etc.*, have been documented. However, the majority of these traditional gas sensors are dependent on an external power source, which limits their portability and raises their maintenance expenses significantly (Wang et al., 2020).

Currently available self-powered solutions include electromagnetic generators (Siddique et al., 2015), fuel cells (Ma et al., 2021), solar cells (Hao et al., 2022), and nanogenerators (NGs). Because of their excellent electrical output performance and straightforward construction procedure, NGs have garnered a lot of attention from researchers in recent years (Kaur and Pal, 2018). NGs are energy conversion devices that fall into two primary categories: piezoelectric nanogenerators (PENGs) based on the piezoelectric effect and triboelectric nanogenerators (TENGs) based on contact electrification and electrostatic induction coupling (Wu et al., 2020).The difference between TENGs and PENGs is that the working principle is different, TENGs generate an unbalanced distribution of charges through the friction of two different materials, and then drive the charge movement to generate electricity with the help of electrostatic induction. When the piezoelectric material in PENGs is deformed by external force, the positive and negative charge centers of the internal lattice generate relative displacement to form a polarized electric field, and the surface of the piezoelectric material will generate a polarized charge. The two electrodes in PENGs induce opposite charges, and when an external circuit is connected, the charge movement generates electricity. In order to provide gas sensors with a continuous self-powered electrical energy supply, NGs can transform low-frequency energy that is widely distributed in the gas environment into useful electrical energy. Examples of this energy include wind energy (Jiang et al., 2021), biomechanical energy (Sardana et al., 2022), and mechanical energy (Lin et al., 2014b; Zhang et al., 2024c). The use of NGs in gas sensors has numerous benefits. First, because NGs are self-powered, gas sensing can function without an external power source. As a result, a variety of NG-based gas sensing systems have been shown to be self-powered, including but not limited to: Hydrogen Sulfide (H_2_S) (Qu et al., 2016; Liu et al., 2023a; He et al., 2018); Organics (Ethanol, Formaldehyde, Acetone) (Lin et al., 2014b; Wang et al., 2015; Wang et al., 2024a; Chang et al., 2022b); Carbon Oxides (CO, CO_2_) (Singh et al., 2023; Zhao et al., 2018; Kim et al., 2019); Nitrogen Oxides (NO_2_) (Su et al., 2020; Su et al., 2018); Nitrogen Hydride (NH_3_) (Wang. et al., 2022d; Wang. et al., 2021a; Veeralingam and Badhulika, 2023). Furthermore, by using self-powered features, NGs-based gas sensing systems can function independently and continuously in remote areas where it may be difficult to obtain an external power source for gas monitoring. Additionally, NGs-based gas sensors offer a smaller sensing system size, making them suitable for applications like precision instruments and wearable devices.

The existing literature reviews the self-powered gas sensors of TENGs in different application scenarios in detail (Zhang et al., 2024a). However, there are few reviews of NGs containing both TENGs and PENGs for self-powered gas sensor applications. This review offers an in-depth analysis of recent research advances in self-powered gas sensors based on NGs, which include TENGs and PENGs. Two kinds of self-powered gas sensors based on NGs are presented, namely, discrete gas sensors and integrated gas sensors. Finally, the discussion concentrates on the progress of the application of self-powered gas sensors based on NGs for the sensing of some representative gases, for which the primary application areas are discussed for each of these gases.

## 2 Classes of nanogenerator-based gas sensors

NGs-based gas sensors are categorized into two modes of operation, which are discrete and integrated. For separated NGs-based gas sensors, the NGs and the gas sensor are two distinct elements. The NGs section is responsible for generating electric power and providing it to the gas sensing section, while the gas sensing section reacts with the gases in the environment through gas-sensitive materials and ultimately realizes the purpose of gas sensing. Among them, the main gas sensing mechanism is resistive gas sensing, and when the physical adsorption, chemical adsorption and catalytic reaction between gas molecules and the surface of gas-sensitive materials occurs, the resistivity of gas-sensitive materials will change accordingly. Since the gas-sensitive material is coupled to the electrodes of the NGs, this change is ultimately reflected in the electrical signal output of the NGs. For example, a self-powered NO_2_ gas sensor based on Pd-modified ZnO/MoSe_2_ nanocomposites, which synthesizes PVA by electrostatic spinning on aluminum foil and fabricates TENGs by using Al-PVA and polyimide film as friction materials. The fabricated TENGs were used as the self-powered power source of the system, which was responsible for powering the Pd@ZnO/MoSe_2_-based NO_2_ gas sensing section, allowing for self-powered NO_2_ gas sensing (Wang et al., 2022c). Similarly in terms of PENGs, MXene/Co_3_O_4_ composite formaldehyde sensors based on ZnO/MXene nanowire arrays driven by PENGs operating at room temperature. Its power supply part is PENGs based on ZnO/MXene nanowire arrays, while the gas sensing part is a MXene/Co_3_O_4_ composite formaldehyde sensor (Zhang et al., 2021).

For integrated gas sensors based on NGs, the NGs and the gas sensor are integrated into one element. In this system the NGs still act as a power source, meanwhile, when the NGs are exposed to the corresponding gas environment, due to the reaction between the gas-sensitive materials in the NGs and the gas, the electrical output characteristics of the NGs will change accordingly, by measuring the change of the relevant electrical parameters, self-powered gas sensing can be achieved. For example, ethanol sensors based on Pd/ZnO nanowire array PENGs, due to the coupling of the piezoelectric effect of ZnO and the gas-sensitive properties of Pd/ZnO, the piezoelectric outputs of these PENGs can be used as both the power source of the system and the sensing signal for ethanol (Lin et al., 2014a). Similarly in the case of TENGs, intermittent airflow-driven TENGs based on ZIF-8/copper foam. Through the contact charging and electrostatic induction properties of the TENGs, and the gas-sensitive properties of the ZIF-8/copper foam material, the TENGs can also be used for both ventilation energy harvesting and formaldehyde concentration detection (Wang et al., 2024b).

Separate gas sensors have relatively independent components, and the two parts are electrically connected through wires and other connections, so that the positions of the NGs and the gas-sensing unit can be adjusted according to the specific needs of the application, which provides better flexibility. While the integrated gas sensor integrates NGs and gas-sensitive materials in a single unit, this integrated structure makes the sensor smaller and also reduces the loss and interference during the transmission of electrical signals.

## 3 Nanogenerators for gas sensing applications

### 3.1 Ammonia

Ammonia sensing technology demonstrates potential for a wide spectrum of applications in several fields. It plays a key function in environmental monitoring, human safety, and food safety (Tornatore et al., 2022).

In environmental monitoring situations, ammonia sensors are designed to detect ammonia concentrations in the environment in real-time to avert potential health problems or to monitor emissions from industrial activities (Han et al., 2024). A two-dimensional niobium carbide MXene/polyaniline sensor driven by TENGs has excellent ammonia sensing performance, and the fastest response time can be as low as 105 s by adjusting the spray volume of two-dimensional niobium carbide MXene nanosheets (Wang et al., 2021c), which provides a new idea for high-performance room-temperature ammonia gas detection.

**FIGURE 1:**
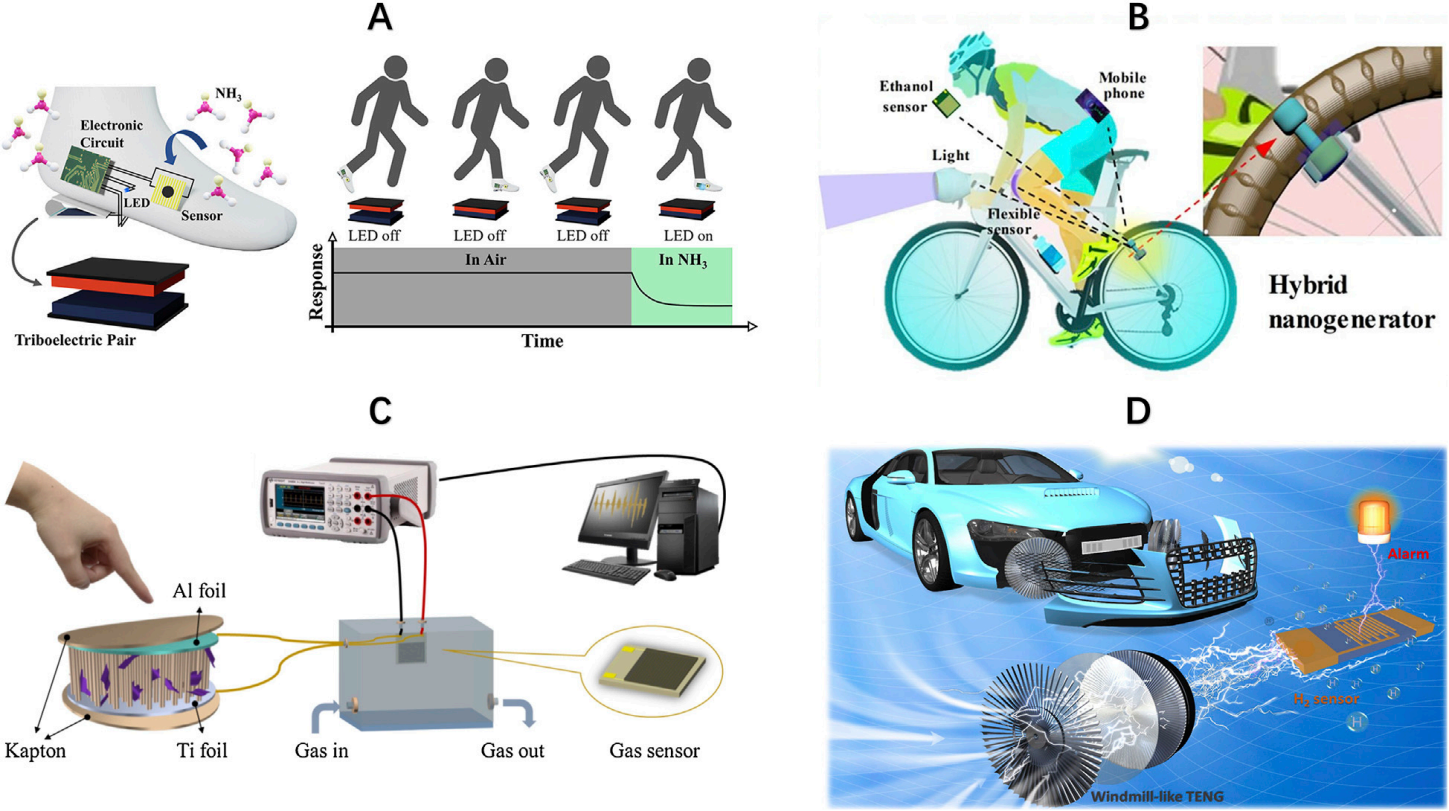
**(A)** Schematic of self-powered ammonia monitoring device embedded in an insole to detect ammonia concentration; reproduced with permission from Sardana et al. (2022). **(B)** Schematic of a hybrid nanogenerator utilizing wheel rotation to generate electricity; reproduced with permission from Wang et al. (2022b). **(C)** Schematic of HCHO sensor driven by PENG; reproduced with permission from Zhang et al. (2021). **(D)** Schematic of windmill-like TENGs utilizing wind energy to power a hydrogen leak detector; reproduced with permission from Jiang et al. (2021).

In addition, self-powered wearable ammonia monitoring devices are becoming a hotspot because they eliminate the dependency on external batteries and human wearability, and automated ammonia detection may be accomplished by embedding self-powered ammonia monitoring devices into insoles (Wang et al., 2021b). As shown in Figure 1A, TENGs based on MXene nanofibers and cellulose acetate nanofibers can convert the mechanical energy generated by the human body when walking into electrical energy, and reach a maximum instantaneous power density of 1,361 mW/m^2^ at a load of 2 MΩ to power the MXene/TiO_2_/cellulose nanofibers heterojunction-based ammonia sensors, and at the same time, the on/off conditions of three different color LEDs are used as the warning signals for exceeding ammonia concentration, realizing the effective construction of a self-powered ammonia detection and warning system (Sardana et al., 2022). In addition to being implanted in insoles, PENGs wearable devices built on flexible MoS_2_ sheets connected to the human body have been proven to harvest mechanical energy from human movement to create electricity to operate room-temperature Au-MoSe_2_ composite ammonia sensors (Zhang et al., 2019). The NGs not only enable long-term autonomous operation of the sensors but also considerably increase the mobility and usefulness of the system, giving a novel solution for the detection of dangerous gasses and human safety areas.

Ammonia sensing technology also plays a significant role in the realm of food safety. Ammonia detection during cold chain transit of food is an essential technique for verifying the quality of perishable food. As the degree of food decomposition advances, the concentration of ammonia emitted from the food will climb dramatically (Ip and Chew, 2010). TENGs wireless gas sensor system may be utilized for the detection of ammonia emitted by pork and seafood rotting. Through the wireless transmission module, the ammonia concentration may be transferred to the user interface in real-time, and the user can determine the quality of the food based on the ammonia concentration. It also retains outstanding stability at high humidity (75%) and low temperature (18°C), which permits the system to be employed in cold chain transportation environments (Cai et al., 2021). In addition to ammonia detection during cold chain transit, it is also vital to detect ammonia emitted from meat at room temperature. A TENGs-driven MXene/CuO ammonia sensor based on latex and PTFE composites successfully realized real-time detection and evaluation of pork spoilage by accurately measuring the ammonia concentration released from pork samples under room temperature conditions during different storage cycles (Wang et al., 2021a). Provides new technical means in the realm of food quality monitoring.

### 3.2 Ethanol

In recent years, ethanol sensors based on NGs technology have shown tremendous potential for creative applications in numerous domains because of their unique self-power supply capacity, notably in human health monitoring, environmental monitoring, and industrial process control (Zhang et al., 2013).

In the realm of human health monitoring, given the harmful impact of alcohol consumption on cyclists’ muscular movement coordination and mental attention, detecting cyclists’ drinking status is an essential aspect of preserving their riding safety (Patel et al., 2010). As shown in Figure 1B, By combining TENGs and electromagnetic generators to form a hybrid nanogenerator, it is possible to generate electricity by utilizing the energy generated by the rotation of the wheels, with the TENGs generating up to 581 V at 15 km/h and the electromagnetic generator contributing 62 V, which support a maximum peak power of 4.6 mW and 1.9 W, respectively. The real-time self-powered monitoring of cyclists’ alcohol consumption levels is accomplished by employing a sensor system based on Ti_3_C_2_T_x_ MXene/Ag driven by hybrid nanogenerators, which considerably enhances cycling safety (Wang et al., 2022b), which is crucial for public safety and human health. In addition to self-generated power utilizing wheel rotation, Pd/ZnO nanoarray-based PENGs can produce electricity from the movement of a person’s finger and accomplish ethanol sensing at room temperature. In terms of performance, when the concentration of ethanol gas exposed to room temperature was changed from 200 ppm to 800 ppm, the piezoelectric output voltage decreased from 0.45 V to 0.25 V, which showed a more obvious voltage response trend (Lin et al., 2014a), and the device provides a new idea for human wearable ethanol detection devices.

In industrial production situations, ethanol is a crucial raw material and a potentially dangerous gas, and its concentration monitoring is necessary to maintain production safety. The positive voltage generated by the TENGs is utilized to reduce the Schottky barrier height of the ZnO NMW Schottky sensor so that the response enhancement to 100 ppm ethanol can reach 139%, which can effectively improve the ethanol responsiveness of the sensor, and at the same time, it is tested that the ethanol sensor is still working stably after the number of treatments is more than 100 times (Meng et al., 2020). This self-powered, high-stability ethanol sensor is still able to work independently and stably in tough industrial production conditions, giving a solid assurance for industrial production.

Self-powered ethanol sensors based on NGs still show significant potential for application in agricultural detection. Fruits and vegetables stored in high concentrations of CO_2_ and low oxygen for a long time will undergo anaerobic respiration and create ethanol, the excessive accumulation will impair the quality. In view of this, the researchers produced TENGs based on silicone/MXene@silicone double dielectric layers, which amazingly have an open-circuit voltage of up to 1160 V under ideal circumstances. Combining these TENGs with a CuO/TiO_2_/Mxene ethanol sensor capable of detecting ethanol down to 0.3 ppm at room temperature without the need for an external power supply and with a fast response/recovery time (16/13s) (He et al., 2024), it provides an innovative solution for quality monitoring of fruits and vegetables.

### 3.3 NO_2_

NO_2_, being a frequent contaminant in industrial emissions, poses a substantial hazard to both human health and the natural environment. Because of this, the development of effective, self-powered NO_2_ detection devices is of special relevance. However, the bulk of NO_2_ sensors now available on the market still rely on battery power (Wang et al., 2021d), which limits the ease of their application to some extent. Therefore, leveraging NGs to provide energy for the sensors is recognized as one of the extremely promising alternatives.

NO_2_ in the air is washed into the soil by rain, which can immediately contribute to the contamination of water and soil, and the detection equipment working outdoors confronts the difficulty of battery life (Pandey et al., 2020). To tackle such challenges, self-powered NO_2_ detecting devices based on TENGs have been created. In a self-powered NO_2_ sensor system constructed based on TENGs coupled with In_2_O_3_/PPy sensors, gelatin, and PLA/PBAT are innovatively employed as the friction layer materials for TENGs, which exhibit excellent electrical output performance with peak and RMS power densities as high as 1,386 mW/m^2^ and 185.35 mW/m^2^, respectively (Zhang et al., 2024b). In addition, the system accomplishes a wide range and high sensitivity detection of NO_2_, and successfully avoids the typical battery-powered technique of frequent replacement and maintenance concerns, thereby assuring the long-term stability of the detection.

The significant quantity of NO_2_ created during industrial production can cause considerable irritation to human eyes and respiratory organs when exposed for a long length of time, hence monitoring of NO_2_ in the industrial production environment is of greatest significance (Jion et al., 2023). However, traditional detection technology still relies on an external power supply, which makes it impossible to fulfill the need for mobility, and nitrogen dioxide sensors operated by NGs successfully make up for this limitation. The performance of TENGs built of weighing paper and PTFE film as friction material is exceptional, which can output peak-to-peak voltage up to 600 V and output power up to 13 mW. Remarkably, the self-powered sensing system comprising these TENGs with In_2_O_3_ nanocubes/SnS_2_ nanoflower NO_2_ sensors achieves a response/recovery time of 45 s/147 s for 50 ppm NO_2_ (Yang et al., 2021), which gives a good sensing performance. It satisfies the pressing requirement for compact and low-power sensors in industrial manufacturing.

### 3.4 Other gases

Formaldehyde concentrations in human exhaled breath can be utilized as a possible marker for various diseases, including, but not limited to, respiratory disorders, neurological diseases, and malignancies (Protano et al., 2022), making the examination of human exhaled formaldehyde particularly significant. The breath-driven TENGs made by Ti_3_C_2_T_x_ MXene and NH_2_-MWCNTs composites may display varied output voltage characteristics when subjected to varying quantities of formaldehyde and can be employed in both power supply and formaldehyde sensors. The TENGs feature a peak open-circuit voltage of 136 V and a peak output power of 27 μW, as well as good performance in formaldehyde detection, including a 35% response rate at 5 ppm, a lower limit of detection down to 10 ppb, with rapid response (51 s) and recovery (57 s) times (Wang et al., 2022a). This makes the device beneficial for practical applications in human inhaled formaldehyde detection and associated illness diagnosis, but its limitation is that it is not flexible and wearable. The PENGs based on ZnO/MXene nanowire arrays also exhibit strong electrical output characteristics, and their output voltages can exceed around 750 mV when measured at a constant load of 10 N and an operating frequency of 6 Hz with great stability. As shown in Figure 1C, This PENGs were proven to drive flexible gas sensors based on MXene/Co_3_O_4_ composites for HCHO detection at room temperature with a response/recovery time of 83 s/5 s for 10 ppm HCHO (Zhang et al., 2021). Address the issue of formaldehyde concentration monitoring devices not being able to wear the challenging.

Hydrogen-powered cars have grown quickly in recent years. However, because hydrogen is a volatile and explosive gas, its safety hazards have grown increasingly apparent (Brzezinska, 2018). Therefore, self-powered hydrogen leakage sensors play an essential role in the field of hydrogen energy vehicle safety. As shown in Figure 1D, Self-powered hydrogen sensing can be accomplished based on impedance-adjustable windmill-like TENGs that can utilize the wind energy from a hydrogen-energy vehicle during driving to power a hydrogen leak detector. The output voltage fluctuates with the state of the Pd/ZnO nanorods hydrogen sensor, which may be directly reflected in the status of the LED indicator (Jiang et al., 2021). This invention not only gives a solid assurance for the safe operation of hydrogen energy vehicles but also highlights the extensive application potential of TENGs in the field of hydrogen safety monitoring.

## 4 Summary and outlook

NGs-based self-powered gas sensors successfully eliminate the limits of standard gas sensors in terms of power supply, exhibiting considerable benefits and potential. This paper introduces NGs-based self-powered gas sensors categorized into separated and integrated types and focuses on the progress of NGs-based self-powered gas sensors for ammonia, ethanol, nitrogen dioxide, and other gases, for which their main application areas, including environmental monitoring, food quality monitoring, human health monitoring, industrial monitoring, and agricultural monitoring are presented, respectively. At present, the application of NGs to gas sensors has been proved to be a feasible solution, which can convert the widely distributed low-frequency energy in the gas environment into useful electrical energy to power the gas sensing system and make up for the shortcomings in the power supply of traditional gas sensors. In the future, via the introduction of new materials, NGs-based gas sensors will be employed to monitor more gases. In addition, at present, nanogenerators still have the problem of low energy efficiency conversion, which limits their application in high-power equipment. In the future, by optimizing the preparation process of NGs, such as using doped piezoelectric materials in the preparation of PENGs and selecting different modification methods according to the properties of selected friction electrodes in the preparation of TENGs, the electrical output performance of NGs is expected to be further enhanced. Doping the piezoelectric material will affect the lattice structure of the material, thereby enhancing the piezoelectric coefficient of the material and improving the electrical output performance of PENGs. A typical modification method for triboelectric layers is surface coating modification, which increases the surface charge density and thus improves the electrical output performance of TENGs. Gas sensors based on NGs will also face various sources of interference, such as high temperatures in industrial environments. The electronic thermal emission rate of the friction layer increases with rising temperature, thereby reducing the charge storage capacity of the friction layer, ultimately leading to a decrease in the output of the TENG. Therefore, discussing how to effectively address the interference of high temperatures on the performance of gas sensors based on NGs is an important research direction for the future.
